# Global Emerging Infections Surveillance Program Contributions to Pandemic Preparedness and Response

**DOI:** 10.3201/eid3014.240305

**Published:** 2024-11

**Authors:** Kathleen E. Creppage, M. Shayne Gallaway, Dara A. Russell, June M. Early, Hunter J. Smith, Aileen C. Mooney, Ashley M. Hydrick, Matthew R. Kasper

**Affiliations:** Defense Health Agency, Silver Spring, Maryland, USA (K.E. Creppage, M.S. Gallaway, D.A. Russell, J.M. Early, H.J. Smith, A.C. Mooney, M.R. Kasper); Fort Eisenhower, Augusta, Georgia, USA (A.M. Hydrick)

**Keywords:** Global Emerging Infections Surveillance Program, GEIS, Department of Defense, DOD, pandemic, pandemic preparedness, pandemic response, COVID-19, respiratory infections, severe acute respiratory syndrome coronavirus 2, SARS-CoV-2, SARS, coronavirus disease, zoonoses, viruses, coronavirus, United States

## Abstract

Since its establishment in 1997, the US Department of Defense (DoD) Global Emerging Infections Surveillance (GEIS) program has provided support for infectious disease pandemic preparedness and response. The GEIS program has shown the value of having a central hub responsible for coordinating a global network of DoD laboratories that conduct surveillance for militarily relevant infectious disease threats. The program has supported the establishment and maintenance of capabilities for collecting, characterizing, and reporting on major infectious disease events, including the COVID-19 pandemic and mpox outbreak. The GEIS program enables the US government to mitigate infectious disease threats to DoD mission readiness and to effectively respond to pathogens worldwide. Continued investment in maintaining the GEIS program and its network is critical for timely detection and response to future emerging infectious disease threats in various populations within locations where gaps in US government or host-nation surveillance might exist.

The mission of the US Department of Defense (DoD) is to provide combat-credible military forces needed to deter war and protect the security of our nation and its allies (*1*). To this end, DoD supports pandemic preparedness and response through its unique global presence and ability to provide rapid response, deployment, and logistical capabilities, thereby complementing and enhancing the capabilities of its civilian counterparts (*1*). As the 2022 National Biodefense Strategy and Implementation Plan notes, infectious diseases ignore borders and can compromise the effectiveness of deployed forces and mission readiness, degrading operational capabilities and mission success (*2*). The COVID-19 pandemic highlighted the substantial burden that disease can place on military populations, which can include mission postponements or cancellations. For example, the aircraft carrier USS Theodore Roosevelt and the guided-missile destroyer USS Kidd both experienced mission interruptions because of onboard COVID-19 outbreaks (*3*,*4*), underscoring the need for proactive infectious disease surveillance and response.

The Global Emerging Infections Surveillance (GEIS) program was established in 1997 in response to the Presidential Decision Directive, National Science and Technology Council 7, on emerging infectious diseases (Figure) (*5*). The directive expanded the DoD’s mission to include support of global surveillance, training, research, and response to emerging infectious disease threats through centralized coordination, improved preventive health programs and epidemiological capabilities, and enhanced involvement with military treatment facilities and United States and overseas laboratories (*5*). The GEIS program’s purpose supports health protection for military forces and supports alignment with other federal agencies. The program’s purpose ensures it is both unique and effective within the DoD because it informs US national biosurveillance and biodefense strategies by enhancing understanding and controlling the effects of emerging infectious diseases among US military service members. When the directive was issued, the DoD maintained 3 core laboratories within the United States and 6 overseas laboratories. GEIS now operates through a more expansive network of strategically positioned military service, Defense Health Agency, and public health partner laboratories across all 6 geographic combatant commands. 

**Figure:**
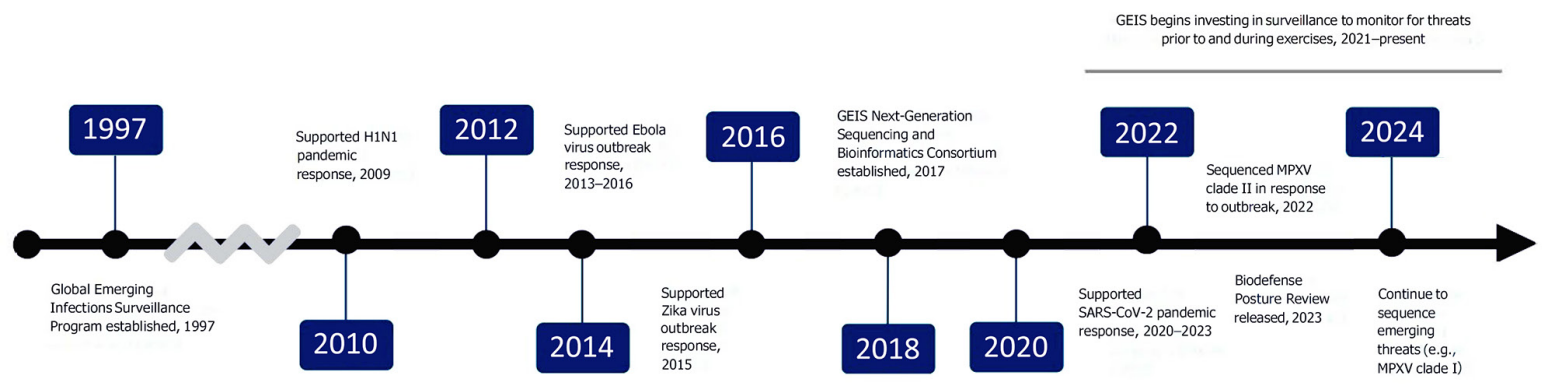
Timeline of key Department of Defense Global Emerging Infections Surveillance program events in support of pandemic preparedness and response, United States. Boxes and text indicate critical activities that occurred on or within specific time points. Time points were selected if multiple activities occurred within a several year period. Surveillance program was established in 1997 and has supported and continues to support multiple pandemic and outbreak responses, beginning with pandemic influenza. Key events in the GEIS program history are indicated where the program office or its partners provided support for infectious disease outbreak response or pandemic preparedness activities, including sequencing of samples to better characterize infectious disease threats as they emerged. GEIS, Global Emerging Infections Surveillance; MPXV, monkeypox virus.

The GEIS program is the DoD’s central coordinating hub for infectious disease surveillance and provides critical program management. The program management component encompasses foundational elements that contribute to rigorous scientific proposal review and thoughtful strategic selection of a surveillance portfolio to maximize efficiency and effectiveness of the GEIS network (GEIS-N) in threat surveillance. The GEIS program also leverages communication modalities to ensure timely information is shared with relevant parties and includes formal reports, peer-reviewed articles, and informal communications.

## GEIS Pandemic Preparedness Timeline of Success

Since its inception, the GEIS program has shown the utility of funding and coordinating infectious disease surveillance. In 1998, the program published a 5-year plan titled Addressing Emerging Infectious Disease Threats: A Strategic Plan for the Department of Defense*,* a DoD strategy for protecting military forces and US citizens from relevant microbial threats (*6*). This initial plan paralleled the 1994 strategic plan of the US Centers for Disease Control and Prevention and its subsequent updates (*7*,*8*). After 1998, the GEIS program’s baseline budget increased for overseas DoD laboratories; the program became the responsible management entity to ensure the execution and return on investment of those funds (*9*–*11*). The GEIS program has continued adapting its strategy for addressing potential threats, and the program and network have matured and evolved to better respond when public health emergencies have surfaced. DoD laboratories have consistently shown their ability to pivot to detect and characterize the next emerging pathogen. Through its baseline, supplemental COVID-19, and Biodefense Posture Review funds, GEIS has invested in activities that generate timely and actionable information regarding ongoing threats, including antimicrobial drug resistance, malaria countermeasure failures, and novel respiratory viruses, as well as supporting several concerning public health emergencies since 2010 (Figure).

In 2009, GEIS partner laboratories detected early cases of novel influenza A(H1N1) and coordinated additional diagnoses, advanced characterization through next-generation sequencing, and reporting of novel influenza cases in >10 countries worldwide (*11*). That same year, partner laboratories were able to detect a concerning number of clinically relevant drug-resistant and unexpected organisms in various parts of Southeast Asia, highlighting the flexibility of the network even in its earliest days. During the 2016 Zika virus infection outbreak, the GEIS program distributed nearly US $2 million to support enhanced Zika virus surveillance activities in 18 countries. Part of that comprehensive response was surveilling mosquito vectors and serum samples from US military personnel for traces of the virus (*12*,*13*). In addition, the GEIS program received and invested millions of dollars in support of SARS-CoV-2 surveillance, including assay development and distribution, laboratory training, diagnostics, sequencing, and phylogenetic analyses. The GEIS program’s effective response to the SARS-CoV-2 pandemic emphasizes the rapid, comprehensive, and agile elements of the program and its network. Since 2018, the GEIS program has supported the detection of >27 novel pathogens by DoD laboratories.

Part of the success of this program and its support for pandemic preparedness and response is attributable to sustaining routine longitudinal regional surveillance, an often understated and underappreciated benefit of the network. GEIS-funded influenza surveillance is a model for how investments in routine activities, capabilities, and infrastructure built a foundation for pandemic preparedness and response. For example, the GEIS program provides funding for the DoD Global Respiratory Pathogen Surveillance Program, a network of sentinel surveillance sites that can respond to infectious disease threats. The pathogen surveillance program supplies the GEIS-N with a diverse, representative sample pipeline used for diagnostic methods and advanced characterization through next-generation sequencing of infectious respiratory pathogens. That network was integral to the DoD response to the COVID-19 pandemic, enabling an influx of samples from military installations across the world, including remote or inaccessible locations.

Microbial genomic sequencing technologies are leveraged across the GEIS-N to provide more comprehensive infectious disease information for pandemic preparedness and response. In 2017, the GEIS program established a consortium of next-generation sequencing and bioinformatics experts from across the DoD to focus on improving the translation of microbial sequence data into public health practice. That consortium, armed with resources, relationships, and expertise in virus characterization, provided support within the DoD at the onset of the COVID-19 pandemic. The GEIS program also coordinated vital statistics data collection and reporting efforts and, in 2021, developed and executed a plan for expanding SARS-CoV-2 genomic surveillance within the Military Health System (*13*). As a result, the GEIS program has maintained a repository of SARS-CoV-2 genomic surveillance data since 2020, with the knowledge that well-resourced, routine surveillance is the backbone of preparing for the next threat. Routine surveillance and timely advanced characterization data generated by GEIS-funded laboratories are also shared with partner nations and facilities that might otherwise lack access to those data. For example, as part of the Makerere University Walter Reed Project’s antimicrobial resistance surveillance program, GEIS-funded partners characterized hypervirulent, multidrug-resistant *Klebsiella pneumoniae* in 4 tertiary healthcare facilities, providing evolution, convergence, and transmission data, which informed clinical decision making and infection control within those facilities (*14*). Much of that information is shared through publicly available databases, such as GISAID (https://www.gisaid.org) and GenBank, to enable analyses outside of the DoD. The GEIS program, along with additional partner laboratories, can rapidly scale-up sequencing capabilities in response to emerging threats by leveraging existing surveillance sites, personnel, and infrastructure.

## Preparing for the Future of GEIS

The GEIS program is poised to support pandemic response efforts through continuous maintenance of a robust structure and through the flexible, scalable capabilities of its network. The infrastructure built around influenza and respiratory disease surveillance enabled a rapid pivot to support response efforts during the COVID-19 pandemic (*15*), which would not have been possible without an existing surveillance platform. As the COVID-19 pandemic declined, GEIS-N partners pivoted their sequencing capabilities to the next known disease of interest: mpox. Despite its rapid emergence and vastly different genome size and complexity compared with SARS-CoV2 (*16*), monkeypox virus samples were sequenced by >3 GEIS-funded partners, demonstrating the readiness and ability of GEIS-N partners to respond to diverse and complex challenges during the immediate post–COVID-19 pandemic era. The GEIS program also looks for opportunities to further invest in advancements in biosurveillance and pandemic preparedness, such as novel surveillance activities contributing to earlier detection and response; solutions for enhanced data collection, storage, and sharing; broad-based agreements that enhance partnerships within the GEIS-N; and partnerships with external agencies that can encourage a whole government approach to preventing, detecting, and responding to infectious disease threats. The GEIS program has also funded projects using wastewater surveillance methods for infectious disease detection and has explored opportunities to further coordinate and broaden expertise in this surveillance domain.

In summary, the success of the GEIS program and its partner network must be sustained; maintaining robust capabilities for future emerging threats is required for DoD’s military readiness, as highlighted in the Biodefense Posture Review released in 2023 (*17*). Through the GEIS program and its network, DoD laboratories are monitoring for known and unknown pathogens (including those that might pose a novel threat), building an information baseline that is imperative for identifying potential threats to military service members and their operations. Continued investment in the GEIS program and its network is critical for timely detection of and response to future emerging infectious diseases in various populations in locations where gaps in US government or host-nation surveillance might exist. 
